# Hydrophobicity and Photocatalytic Activity of a Wood Surface Coated with a Fe^3+^-Doped SiO_2_/TiO_2_ Film

**DOI:** 10.3390/ma11122594

**Published:** 2018-12-19

**Authors:** Luning Xuan, Yunlin Fu, Zhigao Liu, Penglian Wei, Lihong Wu

**Affiliations:** 1 College of forestry, Guangxi University, Nanning 530004, China; xluning@126.com (L.X.); ztzxwpl@163.com (P.W.); lljheng@126.com (L.W.); 2 MOE KEY Laboratory of Wooden Material Science and Application, College of Materials Science and Technology, Beijing Forestry University, Beijing 100083, China

**Keywords:** wood surface, sol–gel method, Fe^3+^-doped SiO_2_/TiO_2_ composite film, photocatalytic activity, hydrophobicity

## Abstract

A Fe^3+^-doped SiO_2_/TiO_2_ composite film (Fe^3+^-doped STCF) was prepared on a wood surface via a sol–gel method to improve its photocatalytic activity and hydrophobicity. The structure of the composite film was analyzed by Fourier Transform infrared spectroscopy (FTIR), X-ray diffraction (XRD), and X-ray photoelectron spectroscopy (XPS). The photocatalytic activity toward degradation of methyl orange and its hydrophobic nature were investigated. The results showed that the composite film was anatase TiO_2_ crystal form, and the addition of Fe^3+^ ions and SiO_2_ enhanced the diffraction peaks for the anatase crystal form. The photocatalytic activity of the wood coated with the composite film was enhanced. The highest degradation percentage was at 1 wt % Fe^3+^ (40.37%), and the degradation ability of the wood towards methyl orange solution was further improved under acidic conditions. In addition, the composite film was hydrophobic, and the hydrophobic property was enhanced as the immersion time in the sol increased. The wood surface coated with Fe^3+^-doped STCF exhibited strong hydrophobicity and photocatalytic activity, which could effectively prevent moisture from adhering to the surface and degrade organic pollutants; thus, the modified wood surface had good self-cleaning function.

## 1. Introduction

Wood is the only renewable resource among the four major materials and is widely used in home furniture, construction, and other fields because of its good aesthetic characteristics and environmental friendliness. However, wood surfaces in actual use are susceptible to interference from external factors, which change their physical and chemical properties. For example, wood is hydrophilic, and after water adheres to the wood surface, the hygroscopicity and dimensional stability of the wood changes leading to defects such as deformation [1,2,3]. In addition, wood is more likely to be attacked by fungi resulting in wood decay [4,5]. Contaminants attached to the wood surface will change the color and even change the chemical structure of the wood surface causing adverse effects. Wood’s own defects can also seriously affect its use, and it is necessary to modify the wood so that self-cleaning surfaces can be achieved. Generally, the modification of wood surface gives it photocatalytic activity and hydrophobicity, which are important topics in modern lumber.

Wood surface modification can be divided into organic modifications, inorganic modifications, and other modifications. For example, surfactants can improve the adhesion of the interface of wood–plastic composites [6,7], and benzylation was applied to improve the properties of wood surface [7]. Wood surfaces processed by alkyl groups have better mold resistance [8]. Moreover, impregnating wood with preservatives [9] and processing the wood surface by acetylation [10] can delay wood degradation by Ultra-Violet light. However, these did not fundamentally improve the resistance of the material against photo-degradation. In addition, chromium-based preservatives are toxic. Recently, the use of new technologies to modify wood has gradually attracted researchers’ attention. The volume expansion coefficient of wood has been investigated by studying the time-dependent zeta potential of the wood surface [11]. Li et al. [12] modified the wood surface using a laser to obtain the desired wood color, and a linear relationship between laser modification parameters and wood color was achieved. Guo et al. [13] built a copper nano-coating on the wood surface using photon curing from which the electrical conductivity of wood was achieved. To obtain more hydrophobic wood, discharging the wood surface via gas (plasma) [14] and dielectric barrier discharge [15] has been described. However, these methods need many external instruments, and the steps were relatively cumbersome. This is not conducive to widespread use. 

The use of inorganic nanomaterials to improve wood surfaces has received widespread attention. Wood surfaces can be treated with inorganic materials such as SiO_2_ and TiO_2_ via a sol–gel method to improve its mechanical strength, thermal stability, dimensional stability, flame retardancy, anti-photodegradability, corrosion resistance, and hardness [16,17,18,19]. Shabir et al. [20] prepared a gel layer on the surface and inside of the wood via a sol–gel method. This hydrophobic layer was used to study the copper ion extraction efficiency of TiO_2_ and SiO_2_ precursors. Man et al. [21] immobilized anatase titanium dioxide on grafted cellulose intercalated montmorillonite and found that this composite could effectively degrade methyl orange molecules. The Mn-doped TiO_2_ supported on the surface of wood-based activated carbon fiber had a highly developed pore structure. Its photodegradation efficiency towards methylene blue under visible light irradiation reached 96% [22]. Goncalves et al. [23] used tetraethyl orthosilicate, octyltrimethoxysilane, and phenyltrimethoxysilane as precursors to prepare TiO_2_ particles on the surface of cellulose fibers via layer-by-layer deposition. The as-prepared fiber had a self-cleaning surface and a reinforcing body—these were used to prepare a polymer material that combined cellulose-based composite materials and polymers. This polymer material had good hydrophobicity and stability against irradiation. Therefore, with reasonable design, a film could be constructed on the wood surface so that it has both hydrophobic and photocatalytic properties leading to self-cleaning features. Technology needed to prepare photocatalysts by doping inorganic materials with metal ions has been well developed, but there are a few reports on their applications on wood surfaces. Here, A Fe^3+^-doped SiO_2_/TiO_2_ composite film (Fe^3+^-doped STCF) was coated on the wood surface via a sol–gel method. The Fe^3+^ ions were added to further improve the photocatalytic activity and hydrophobicity of the wood surface resulting in a self-cleaning function.

## 2. Materials and Methods

### 2.1. Materials

The wood samples were Eucalyptus taken from Liangfengjiang National Forest Park, Nanning, Guangxi, China. After the wood samples were air-dried (water content 15%), they were processed into specifications of 25 (L) × 25 (R) × 3 mm (T), washed in an ultrasonic bath for 15 min, then dried. The organic solvents and chemical reagents include butyl titanate (Damao Chemical Reagent Factory, Tianjin, China), tetraethyl orthosilicate (Kelon Chemical Reagent Factory, Chengdu, China), anhydrous ethanol (Damao Chemical Reagent Factory, Tianjin, China), nitric acid (Jinshan Chemical Reagent Co., Ltd., Chengdu, China), acetylacetone (Kelon Chemical Reagent Factory, Chengdu, China), iron nitrate nonahydrate (Chemical Reagent Co., Ltd., Nanjing, China), and ultrapure water. Unless otherwise specified, they were not subjected to further treatment before use.

### 2.2. Preparation of Composite Films

The tetraethyl orthosilicate, anhydrous ethanol, ultrapure water, and acetylacetone were mixed at a ratio of 1:6:8:0.05, and nitric acid was then added to adjust the pH to 2–3; the mixture was heated and stirred at 26 °C for future use. The composite film made of pure silica gel was referred to as the SCF (silicon composite film). The tetrabutyl titanate, anhydrous ethanol, ultrapure water, acetylacetone, and nitric acid were mixed in two steps at a ratio of 1:10:2:0.5:0.2. Specifically, tetrabutyl titanate was first mixed at 2/3 of anhydrous ethanol and acetylacetone forming solution A. After vigorously stirring solution A for 5 min, solution B was prepared by mixing 1/3 anhydrous ethanol, ultrapure water, and nitric acid. The mixed solution was stirred for 30 min. The composite film prepared from pure titanium was referred to as TCF. The SCF and TCF as prepared above were mixed, and different mass fractions of iron nitrate nonahydrate (serving as precursor materials for iron ions) were added into the mixture. The mixed sols with different doping amount of Fe^3+^ ions were heated and stirred for 30 min and kept for later use. The doping amounts of iron nitrate nonahydrate were 0.05, 0.1, 0.5, 1, 2.5, 5, and 10 wt %, respectively. The samples were referred to as STCF0.05–STCF10.0. The prepared sols were aged for 3 h before coating. The dried wood samples were immersed in the sols for 3 h after aging the as-prepared sols. After impregnation, the wood samples were aged at room temperature for 12 h so that a uniform film formed on the wood surfaces. The wood samples with films were then placed in a drying oven at 103 °C for 6 h forming composite films on the wood surfaces. In addition, SCF, TCF, and TCF doped with different amounts of Fe^3+^ ions (STCF-1 and STCF-10) under 800 °C calcination were similarly prepared. These films were also investigated by FTIR and XRD. These films were compared with the Fe^3+^-doped STCF to further characterize its chemical structure and crystallization properties. Composite films with immersion times of 6 h and 8 h were prepared under similar conditions to investigate the hydrophobic properties.

### 2.3. Characterization of STCF

#### 2.3.1. Microscopic Morphology and Structural Characterization of Wood Surface

The surface morphologies and elemental compositions of the samples were examined by S-3400N scanning electron microscopy (Hitachi, Ltd., Tokyo, Japan) and by PV8200 energy dispersive energy spectrometry (Hitachi, Ltd., Tokyo, Japan). A gold foil (Au) layer was sputtered on the surface of the sample to improve conductivity before observation.

The XRD analyses used a Rigaku SmartLab X-ray diffractometer (Rigaku Corporation, Tokyo, Japan) with Cu Kα (λ = 1.5406 Å) as the radiation source scanning at 2°/min with 2θ = 15–85°, respectively. The acceleration voltage and acceleration current were 40 kV and 30 mA, respectively. The XRD data were compared with the JCPDS database to obtain the crystal phase, and the main parameters of the unit cells were calculated by related formulas.

The chemical structure of the groups in the composite films was analyzed by Nicolet iS 50 Fourier transform infrared spectroscopy (Rigaku Corporation, Tokyo, Japan). The scanning range of the instrument was 4000 cm^−1^–400 cm^−1^, and the resolution was 32 cm^−1^. Pellets were used for FTIR analysis. The sample for FTIR characterization was prepared by scraping off the film from a wood substrate with a stainless-steel knife. The scraped sample powder was passed through a 100-mesh sieve, mixed with potassium bromide, and ground in a mortar to form a homogeneous mixture. The mixture was placed in a cylindrical mold (Tuopu Instrument Co., Ltd., Tianjin, China) with a diameter of 13 mm and pressed at 200 MPa for about 1 min to form a colorless transparent pellet.

The XPS test was performed on an Escalab 250Xi (Rigaku Corporation, Tokyo, Japan), Thermo instrument using a monochromatic Al target (hυ = 1486.6 eV) as the X-ray source with a spot size of 500 μm. The full spectrum scan range was 0–1400 eV with narrow area scans of C1s, Si2p, Ti2p, and Fe2p. The calibration was performed by setting the binding energy of the indefinite carbon (C1s) to 284.8 eV. The data measured by XPS analysis was compared with the data in Handbook of X-ray Photoelectron Spectroscopy to determine the valence states and binding modes of the elements.

#### 2.3.2. Characterizations on Photocatalytic Activity

The photocatalytic activity of the Fe^3+^-doped STCF was evaluated by the photodegradation ability toward methyl orange solution. The pure ethyl orange solution, eucalyptus sample without composite film coating, and iron-free composite films were prepared for comparison. The experimental variables were the Fe^3+^ doping level and the pH of the initial solutions.

**Figure 1:**
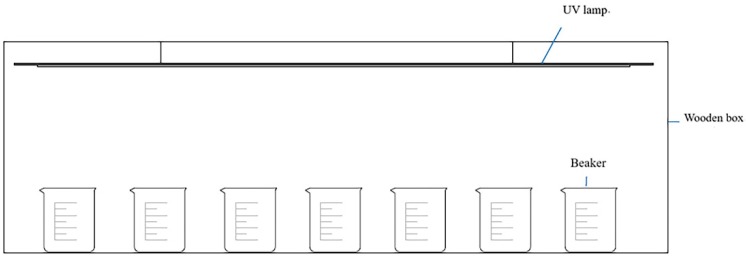
Device design for photocatalytic experiments.

The photocatalytic reaction was carried out using a custom device. The samples were immersed in a beaker containing 100 mL of methyl orange (Jinshan Chemical Reagent Co., Ltd., Chengdu, China) solution (10 mg/L). The ultraviolet irradiation source was 20 cm from the surface of the methyl orange solution (Figure 1). Before irradiation, the reaction solution was kept in the dark and stirred for 30 min to establish the adsorption and desorption equilibrium between the samples to be tested and the methyl orange solution. During the photoreaction, the wood samples coated with the composite films were irradiated with a UV lamp (14 W, 365 nm) (Feiyang Instrument Co., Ltd., Jiangyin, China) under magnetic stirring, and the reaction was performed in air under room temperature. The concentration of the methyl orange supernatant was measured after 2 h of reaction. The concentration of methyl orange was characterized by UV absorption intensity corresponding to the maximum absorption wavelength of methyl orange at 463 nm. The concentration of the residual methyl orange (η) was calculated after measuring with SP-75 visible spectrophotometer (Shanghai Spectrometer Co., Ltd., Shanghai, China) using the equation

(1) $$\eta =C÷C_{0}\times 100\%$$

where *C*_0_ is the initial concentration of methyl orange aqueous solution, and *C* is the concentration of methyl orange solution after ultraviolet light irradiation. 

#### 2.3.3. Characterizations on Hydrophobicity

The hydrophobicity of the sample was evaluated by measuring the water contact angle (WCA). The measurements used a KRÜSS DSA100 contact angle measuring instrument (KRÜSS GmbH, Hamburg, Germany) with a temperature of 18 ± 2 °C and a humidity of 65 ± 20%. The 5 μL of water was placed on the surface of the wood for 10 s. The contact angle was then measured. Five positions were measured on the surface of each sample, and the average value was taken as the contact angle.

## 3. Results and Discussion

### 3.1. Surface Morphology

**Figure 2:**
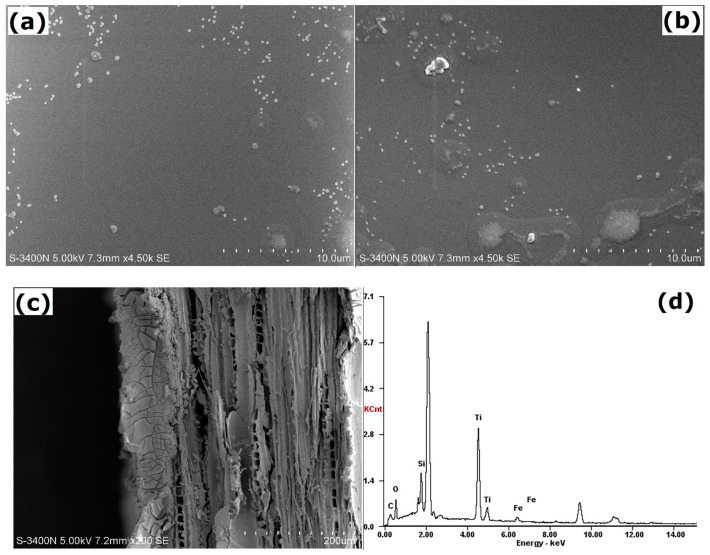
SEM images of (**a**) STCF, (**b**) STCF doped with Fe^3+^, (**c**) the joint surface of wood and composite film, and (**d**) the corresponding EDAX spectrum of STCF-1.

The morphologies of the STCF with and without Fe^3+^ doping are shown in Figure 2. White convex nanoparticles were observed in both the composite films with and without doping indicating that the silicon–titanium substances in the composite films existed as nanoparticles. More nanoparticles were observed when Fe^3+^ was added. Large white particles were formed, and the surface of the composite film became rougher. This might explain why the newly added Fe^3+^ did not react with the silicon–titanium precursor. Instead they might exist as ions or they might agglomerate with the nano-scale titanium precursor—this indirectly affects the overall continuity of the composite film leading to a rougher surface. The composite film was only coated on the surface at the joint of the wood surface and the composite film. It did not penetrate into the interior of the wood without destroying the original structure inside the wood. The thickness was ca. 80–100 nm.

The basic composition of the nanocoating was further analyzed by EDAX (Figure 2d). The peak of the unlabeled element is Au from the conductive layer. The carbon and oxygen are from the wood. After surface treatment, strong peaks are seen for titanium and silicon indicating that silicon and titanium were the base of the composite film. Iron was also observed, and the nano-spherical protrusions on the surface were composed of iron.

### 3.2. Chemical Structure

**Figure 3:**
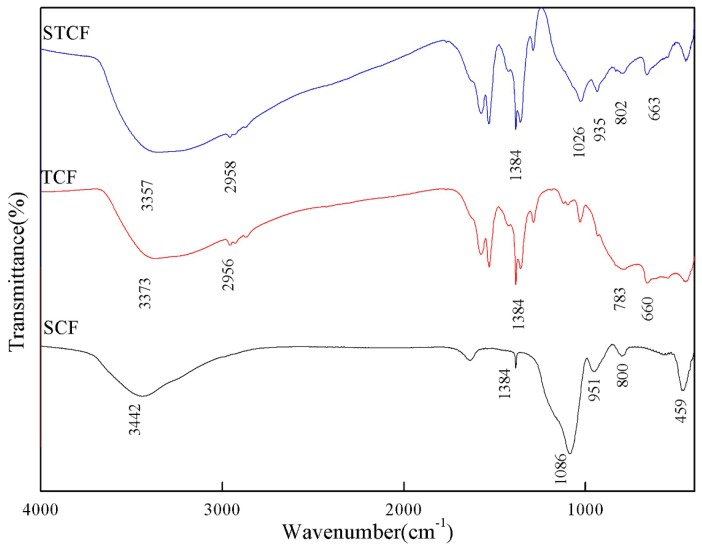
FTIR spectra of SCF, TCF, and STCF.

Figure 3 and Table 1 shows the FTIR spectra of SCF, TCF, and STCF. The FTIR spectrum of SCF (a) had peaks from 3600–3000 cm^−1^. These broad absorption bands were mainly due to the combination of the vibration of Si–OH and H_2_O molecules. Specifically, the –OH group vibrated at ca. 3400 cm^−1^, and the peaks in 3500–3000 cm^−1^ were attributed to molecular water molecules bound to each other or to molecular Si–OH groups [24]. The strong absorption peak at 1384 cm^−1^ was attributed to the symmetrical stretching vibration of NO^3−^ ions [25] which was the residues of nitric acid that were not completely reacted. The peaks at 1086 cm^−1^ and 800 cm^−1^ correspond to the asymmetrical stretching vibration of the Si–O–Si bond and the symmetrical stretching vibration of the Si–O–Si ring structure, respectively. The weak bending vibration of the Si–O–Si bond is seen at 459 cm^−1^. The absorption peak at 951 cm^−1^ might be due to the interaction of Si–C and Si–OH [24,26]. The FTIR spectrum of TCF (b) was more complicated. Except for the absorption peaks of –OH and NO^3−^ groups, the absorption peaks at 2850 and 2958 cm^−1^ were attributed to the C–H stretching vibrations in –CH_2_ and –CH_3_ groups, respectively [27]. The two peaks at 783 cm^−1^ and 660 cm^−1^ were attributed to the weak and medium vibration peaks of Ti–O–Ti in titanium dioxide, respectively [28]. The FTIR spectrum of STCF was similar to those of SCF and TCF. The peaks for the absorption of the asymmetrical stretching vibration of Si–O–Si bond and the symmetrical stretching vibration of Si–O–Si ring structure shifted to low wavenumbers, which might be due to the interaction of networked Ti–O–Ti and Si–O–Si [29]. The medium-intensity vibration peaks of Ti–O–Ti shifted to high wavenumber. An absorption peak at 935 cm^−1^ was also found in the FTIR spectrum of STCF. This is the vibration peak of the Ti–O–Si bond [28]. This indicated that the SiO_2_ and TiO_2_ networks have reacted with each other and were crosslinked.

**Figure 4:**
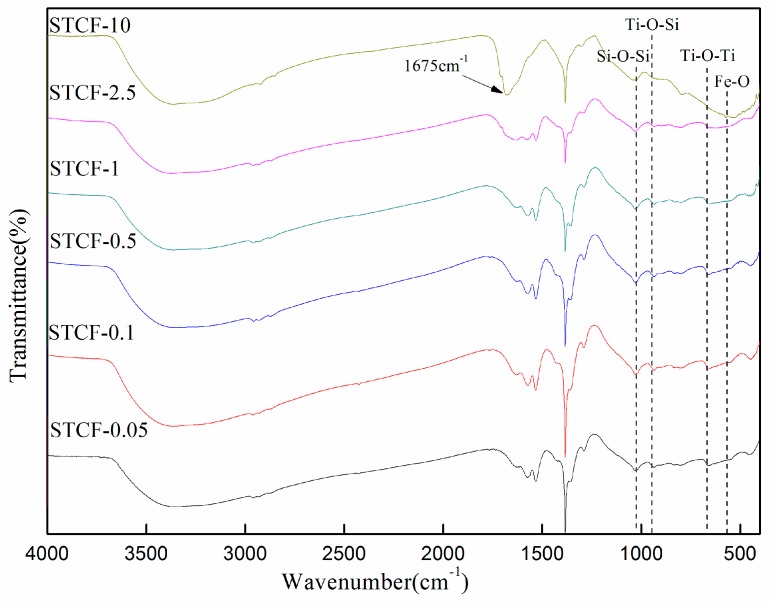
FTIR spectra of STCF with different amounts of Fe^3+^ doping.

Figure 4 shows the FTIR spectra of STCF with different doping amounts of Fe^3+^ ions. The infrared spectra of STCFs were similar when the doping amount of Fe^3+^ ions was below 2.5 wt %. Main groups such as Ti–O–Ti, Si–O–Si, and Ti–O–Si could be clearly distinguished. The adsorption band at 575 cm^−1^ was assigned to Fe–O vibration. A new absorption peak appeared at 1675 cm^−1^ when the doping amount was 10 wt %. This strong peak was attributed to C=C–C=O–. It indicated that some structures like ketene compounds might be generated in the preparation process of STCF-10. Moreover, the weak vibration peak associated with the Ti–O bond was hardly visible, and the peak intensity of the Si–O bond was also reduced. 

### 3.3. Crystal Structure

**Figure 5:**
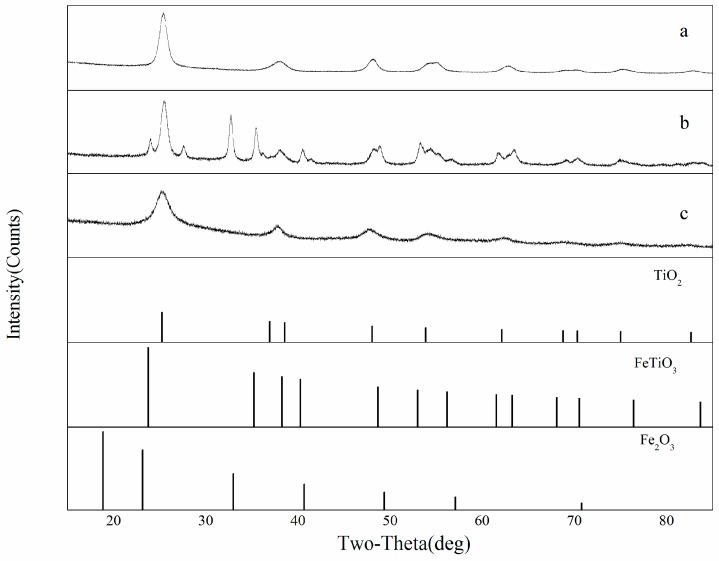
XRD patterns of STCF with different doping amounts of Fe^3+^ ions as well as under different treatment temperatures: (**a**) STCF-1 under 800 °C calcination, (**b**) STCF-10 under 800 °C calcination, and (**c**) STCF-10 dried at 103 °C.

Figure 5 shows the XRD patterns of STCF with different treatment temperatures and different amounts of Fe^3+^ doping. All three samples have TiO_2_ diffraction peaks that belong to the anatase crystal form (2u = 25.28, 37.80, 48.05, JCPDS#21-1272). In addition, the FeTiO_3_ peak (2u = 23.79, 35.25, 38.29, JCPDS#29-0733) was also observed in the XRD pattern of STCF-10 (with a doping amount of 10 wt % and a calcination temperature of 800 °C). No diffraction peaks of independent iron oxides were observed. However, the crystal phase of FeTiO_3_ was not observed in the XRD pattern of STCF-1 (curve a). This might be due to the very small doping amount of Fe^3+^ ions, which was insufficient to combine with the titanium ions thus making it difficult to detect the crystal phase [30]. In the case of samples with the same doping of Fe^3+^, but with different treatment temperatures, only the diffraction peaks of TiO_2_ were observed in the pattern of the STCF-10 sample dried at 103 °C (curve c). The FeTiO_3_ crystal phase formed under high temperature calcination did not appear. This was presumably because the drying temperature was not high at the time of film formation, and this could not meet the requirements for FeTiO_3_ crystallization [31]. In addition, the radius of Fe^3+^ (0.063 nm) is slightly smaller than the radius of titanium ions (0.068 nm), which further proved that Fe^3+^ ions existed as ions in the titanium ion lattice of the SiO_2_/TiO_2_ composite film [32]. This is consistent with previous literature [31] showing that Fe^3+^ ions were uniformly distributed in the crystal structure of TiO_2_. 

**Figure 6:**
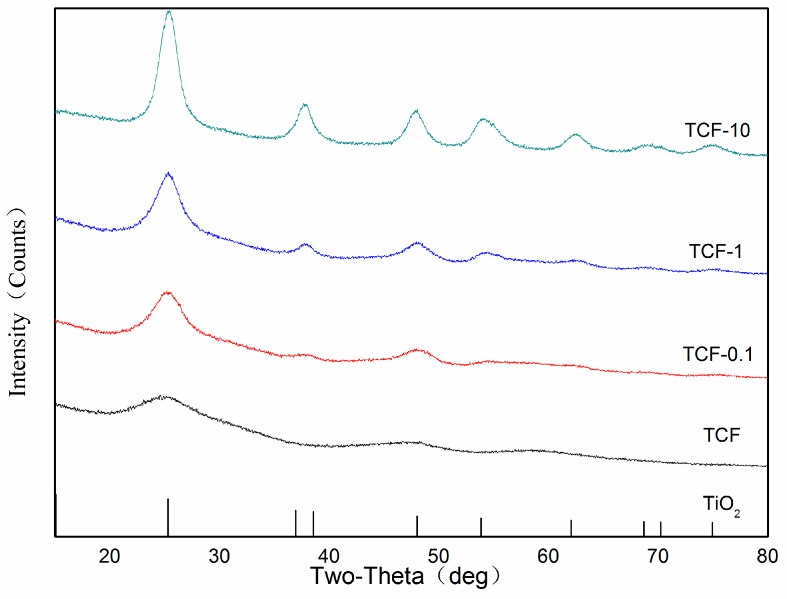
XRD patterns of TCF with different Fe^3+^ doping.

Figure 6 displays the XRD patterns of TCF with different Fe^3+^ doping amounts. All diffraction peaks could be identified as the anatase phase of TiO_2_ (JCPDS#21-1272). No significant characteristic peaks for iron oxides were detected in the samples containing iron suggesting that no significant iron segregation occurred [33]. This might be because the iron content in the sample was below the detection limit of the technique or because the Ti(IV) ions were replaced by Fe(III) ions that entered the lattice of TiO_2_ (IV) [34,35].

Table 2 shows the diffraction angles of the different peak faces of TiO_2_ and the half-width of the interplanar spacing in TCF with different doping amounts of Fe^3+^ as well as the parameters of the standard anatase TiO_2_. The diffraction angle (2θ) and the mirror interplanar spacing d(Å) generated when the amount of Fe^3+^ ions doping was small were closer to the parameters of the tetragonal system of the standard anatase TiO_2_ phase. The crystallite size of different samples could be determined by the Debye–Scherrer equation

(2) D=k×γ÷βcosθ

Here, *D* is the crystallite size in the normal direction caused by the diffractive crystal plane; *θ* is the grazing angle (semi-diffraction angle); *λ* is the wavelength of the incident radiation; and *K* is the Scherrer (0.89). 

The crystallite sizes of the anatase peaks of all the samples are shown in Table 3. The crystallite size of TiO_2_ in TCF doped by Fe^3+^ ions decreased with doping amount indicating that a slight lattice distortion occurred in the anatase structure. The decrease in crystallite size might be caused by the substitution of part of the Ti(IV) lattice positions by Fe(III) cations resulting in crystal defects [36]. The anatase TiO_2_ belongs to the tetragonal system (the unit cell parameters a = b ≠ c), and the diffraction peaks of the (101) and (200) planes were used to calculate the unit cell parameters of the anatase TiO_2_ in different composite films [37].

(3) $$\frac{1}{d^{2}}=\frac{\left(h^{2}+k^{2}\right)}{a^{2}}+l^{2}+c^{2}$$

Clearly, a larger D value and a smaller FWHM value indicates better film crystallization. These observations suggest that the crystallite size of the anatase TiO_2_ thin film as well as the half-peak width decreased with increasing doping amounts of Fe^3+^ ions. This suggests that the doping of Fe^3+^ ions microscopically destroyed the crystal structure of the titanium ions and reduced its crystallinity.

**Figure 7:**
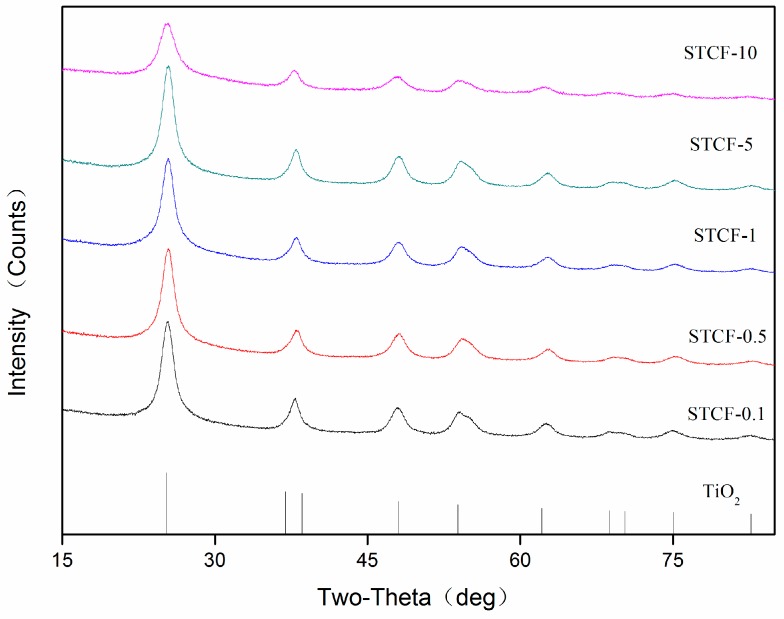
XRD patterns of STCF with different Fe^3+^ doping.

Figure 7 shows the XRD patterns of STCF with various doping amounts of Fe^3+^. Only the diffraction peaks of TiO_2_ were observed in the patterns for all of the composite films dried at 103 °C; no silicon and iron diffraction peaks were detected. This is because the silicon dioxide did not form crystals in the composite films but was present as an amorphous substance. Moreover, the content of doped Fe^3+^ was very low and might be uniformly dispersed in the TiO_2_ particles. Versus the XRD patterns of the composite films without addition of SiO_2_ in Figure 6, the diffraction peak intensities of the composite films after adding SiO_2_ were greatly increased—this will be specifically calculated below. The grain size of the (101) plane of the anatase crystal form was used as a reference in the calculation.

The grain sizes of the catalyst were characterized by the Debye–Scherrer equation as shown in Table 4. The unit cell size of TiO_2_ was 9.007 when the doping amount of Fe^3+^ ions was 0.1 wt %. With increasing Fe^3+^ doping, the unit cell size first increased and then decreased. A maximum value (9.091) was achieved when the doping amount was 0.5 wt %. This is probably because the addition of Fe^3+^ ions could promote the formation of anatase TiO_2_ when the doping amount of Fe^3+^ ions was less than 0.5 wt %. This is a synergistic promotion effect. When the Fe^3+^ doping gradually increased, the iron ions were dispersed into the gel network on the ion-scale range. This might cause disorder on the growth structure of TiO_2_ grains during drying [38]. Some Fe^3+^ entered the TiO_2_ lattice and replaced Ti^4+^ ions to decrease the cell size and inhibit the growth of TiO_2_ grains. The Figure 7 suggests that when the doping amount of Fe^3+^ ions was 10 wt %, the peak intensity of all anatase TiO_2_ decreased, and the full width at half maximum (FWHM) of the peaks also decreased; no diffraction peaks for Fe^3+^ ions were observed. There are three possible reasons for this rationale: (1) the doping amount of Fe^3+^ ions were too small to form an independent oxide phase; (2) the drying temperature was too low to form a crystal phase of iron oxides; (3) in the presence of TiO_2_, the iron ions preferentially entered the TiO_2_ lattice to replace the Ti^4+^ ions instead of forming their own crystalline phase. Versus the XRD patterns of the composite films without SiO_2_, the diffraction peak intensities of the composite films with SiO_2_ were greatly increased. The grain size of the (101) plane of anatase TiO_2_ increased by 4.2, 17.5, and 19.02% for 0.1, 1.0, and 10.0 wt % Fe^3+^, respectively. This indicated that the addition of SiO_2_ can promote the growth of crystalline anatase TiO_2_. Upon comparing the unit cell parameters of TiO_2_ in STCF with different doping amounts of Fe^3+^ ions, we note that a = b ≠ c because anatase TiO_2_ is tetragonal. In addition, the value of c generally decreased as the amount of Fe^3+^ ion doping increased indicating that it did not follow the c-axis orientation perpendicular to the surface of the substrate [39].

### 3.4. Distribution of Elements

**Figure 8:**
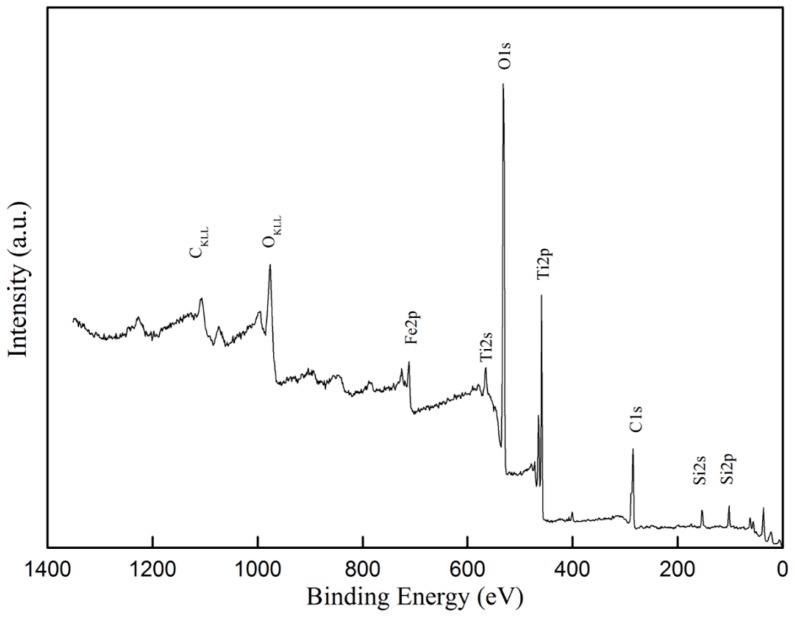
Full XPS spectrum of STCF-1.

**Figure 9:**
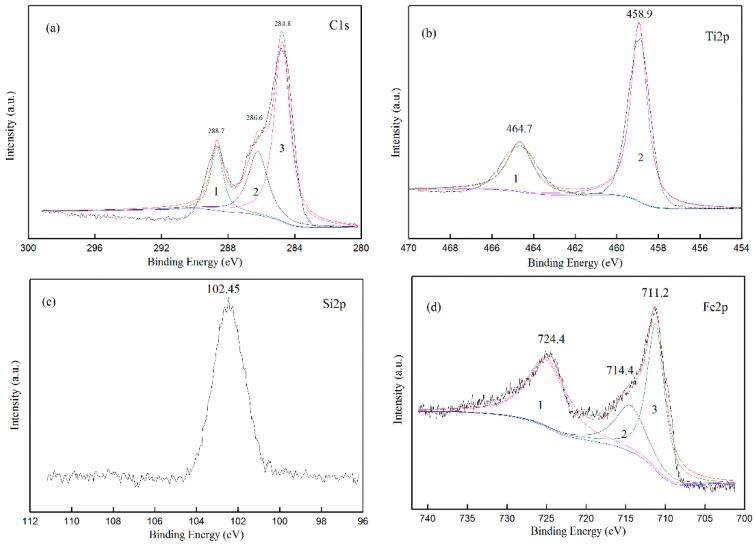
XPS spectra of Fe^3+^-doped STCF: (**a**) C1s; (**b**) Ti2p; (**c**) Si2p; and (**d**) Fe2p.

XPS evaluated the chemical state of all elements on the surface of the composite film. The surface composition and chemical state of STCF-1 was analyzed in Figure 8, which showed that the composite film mainly contained Si, Ti, O, Fe, and C elements. Figure 9 shows the narrow-area X-ray photoelectron peaks of C1s, Ti2p, Si2p, and Fe2p. The background was subtracted using Shirley’s method, and the results were fitted using XPS PEAK41 software. The XPS spectrum of C1s consisted of three peaks as shown in Figure 9a. The first peak could be classified as a –COOH bond via literature data. The second peak was assigned as C–OR—this is the unreacted alkoxy group during the reaction. The third one is the peak for the C–C group [40]. Figure 9b shows the spectrum of Ti2p in the composite film. The two peaks were symmetric indicating that the fitting results of the peaks were correct. By comparing the XPS binding energy standard map, the two spin-orbital components of the Ti2p peak (2p1/2 and 2p2/3) were well deconvolved by two peaks at 464.7 and 458.9. The calculated peak area of the two curves was 2.33, and the distance between the peaks was 5.53 eV. These values and the sharp features of the Ti2p peak in the XPS spectrum indicated that [41] all titanium ions in the composite film had a valence state of Ti^4+^ [42]. Therefore, all titanium ions were present in the form of TiO_2_ in the STCF-1. The Si2p peak in Figure 9c corresponds to the 2p peak of SiO_2_ in the XPS standard manual indicating that all of the silicon ions in the composite film were in the form Si^4+^ in SiO_2_. Figure 9d shows the regio n of Fe2p peaks in the XPS spectrum, and three peaks were obtained by fitting analysis. The two peaks identified as 1 and 3 could be well deconvolved by two curves (about 724.4 and 711.2) [43] indicating that the two peaks were the spin-orbital peaks of Fe2p1/2 and Fe2p3/2 in Fe_2_O_3_. Therefore, in the STCF-1, some doped Fe^3+^ combined with oxygen and was present as Fe_2_O_3_ in the composite film.

### 3.5. Analysis on the Photocatalytic Activity

#### 3.5.1. Effect of Doping Amount of Metal Ions on Photocatalytic Activity

**Figure 10:**
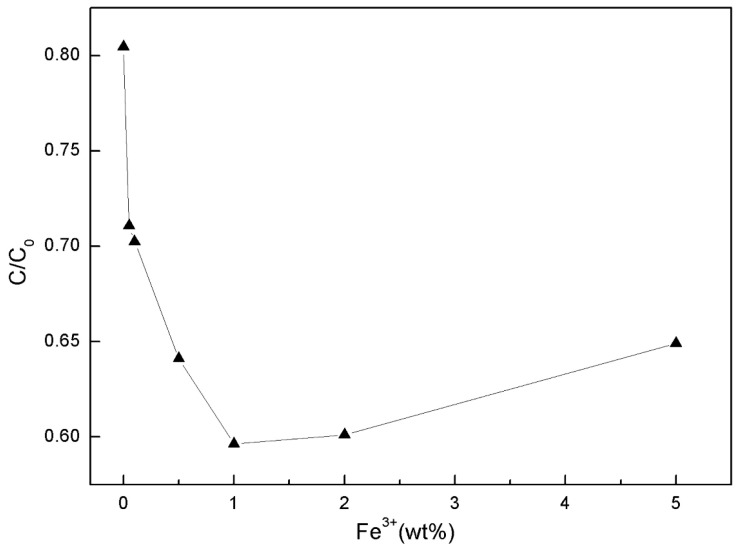
Effect of Fe^3+^ doping on the degradation of methyl orange by STCF.

The degradation of methyl orange by STCF with different doping amount of Fe^3+^ ions under irradiation of a 30 W UV lamp for 2 h is shown in Figure 10. Previous experiments showed that the degradation of methyl orange by the wood was 1.46% after a UV irradiation of 2 h (only 0.16% higher than the degradation of the solution itself). This was negligible and indicates that wood itself can only weakly degrade organic pollutants. The degradation was 19.55% when the wood was coated with the Fe^3+^-free STCF—this was greatly improved versus the wood itself. The degradation was highest (40.37%) when the doping amount of Fe^3+^ increased to 1 wt %. The degradation decreased when the Fe^3+^ doping increased to 2.5 wt %. These findings and the FTIR showed that there was no independent phase for iron-related oxides. Doping would lead to a smaller grain size, and we concluded that the photocatalytic activity of the composite film was modulated via Fe^3+^ doping [44,45].

#### 3.5.2. Effect of pH on Photocatalytic Activity

**Figure 11:**
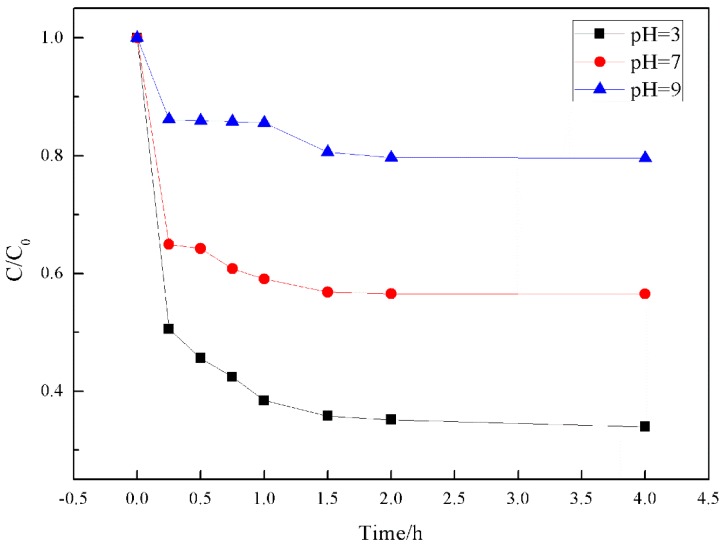
Effect of pH on the degradation of methyl orange by STCF.

The structure and color of methyl orange changed with pH. Thus, the degradation of methyl orange was studied under different pH values to determine suitable conditions for its degradation. Figure 11 shows that methyl orange was more likely to reach adsorption equilibrium under acidic conditions. The efficiency of the catalytic degradation under alkaline conditions was low. In terms of adsorption, the chemical adsorption of methyl orange onto TiO_2_ played the most important role. TiO_2_ is an amphoteric oxide, which forms a titanium alcohol bond in an aqueous solution. At different pH values, the surface of the TiO_2_ catalyst exhibits the following reactions [46]

(4) $$TiOH+H^{+}⇌TiOH^{2+}$$

(5) $$TiOH+OH^{-}⇌TiO^{-}+H_{2}O$$

The isoelectric point of TiO_2_ is pH_zpc_ ≈ 6.8. When the pH of the solution was larger than pH_zpc_, the surface of TiO_2_ was positively charged resulting in TiOH^2+^. At lower pH, the surface of TiO_2_ was negatively charged forming TiO^−^. At low pH, the surface of TiO_2_ was positively charged and attracted negatively charged methyl orange—this increased the adsorption. The surface was negatively charged at high pH, and the adsorption of negatively charged methyl orange was reduced due to Coulombic repulsion. Similar conclusions were obtained when Fan et al. studied the absorption of azo dyes [46]. The pH affected the electrical properties of the surface of the catalyst and the group electrical properties of the dye molecules. The adsorption amount was high at pH = 3.0, but negligible at pH = 9.0.

### 3.6. Analysis on Wetting Performance

**Figure 12:**
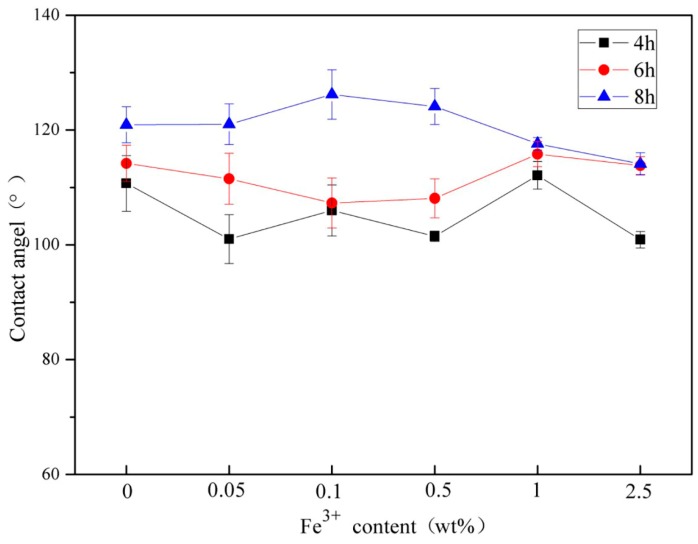
Wetting performance of composite films coated on wood with different Fe^3+^ doping as well as with different immersion times.

The Figure 12 and Table 5 above shows the contact angles of wood samples coated with STCF with different Fe^3+^ doping. The contact angles of wood samples with different sol impregnation times are also shown. The contact angle is most intuitive manifestation of wetting performance. Each horizontal row in the figure shows the contact angle of wood surface with different Fe^3+^ doping levels. The contact angle has no specific trend as the Fe^3+^ doping gradually increases. This indicates that the amount of Fe^3+^ doping had no direct impact on the wetting performance of the composite film. Each longitudinal row shows the contact angle obtained after the wood was immersed in the sol with different times and the same doping amount. The contact angle markedly increased as the immersion time increased indicating that the immersion time was positively correlated with the contact angle of the wood surface. The contact angle reached 126.2° with an impregnation time of 8 h, which indicated that the wood could become hydrophobic via this sol–gel method using low surface energy substances.

## 5. Conclusions

The Fe^3+^-doped STCF was prepared on the surface of wood via a sol–gel method. This improved the photocatalytic and hydrophobic properties of the wood surface and resulted in a self-cleaning function. Structural analysis of the composite film showed that a new Si–O–Ti bond had been formed indicating that the networked silicon dioxide was crosslinked with titanium dioxide. Only the crystalline structure of TiO_2_ with anatase crystal was found in the composite film. The addition of SiO_2_ and Fe^3+^ ions enhanced the crystallinity of anatase TiO_2_ as well as the photocatalytic activity. The silicon and titanium elements in the composite film existed as silicon dioxide and titanium dioxide. Some of the iron ions were present as Fe_2_O_3_. The photocatalytic activity of the composite film towards the degradation of methyl orange solution reached a maximum value of 40.37% when the Fe^3+^ ion doping was 1 wt %. The degradation ability of the composite film was further improved under acidic conditions. Analysis of the wood’s wetting performance showed that the treated sample exhibited a hydrophobic property, which increased with immersion time.

## Figures and Tables

**Table 1:** Wavenumbers of absorption peak for main groups.

Wavenumbers	Corresponding Groups
3400 cm^−1^	–OH
3500–3000 cm^−1^	Si–OH&H_2_O
1384 cm^−1^	NO^3-^
1086&800&495 cm^−1^	Si–O–Si
783&660 cm^−1^	Ti–O–Ti
935 cm^−1^	Ti–O–Si

**Table 2:** Parameters of the diffraction peaks of TCF with different Fe^3+^ doping.

Sample	TiO_2_ Prepared in This Work				JCPDS 21-1272	
	(h k l)	2θ (°)	d (Å)	FWHM	2θ (°)	d (Å)
0.1 wt % Fe–TiO_2_	(1 0 1)	25.210	3.5296	0.932	25.281	3.5200
	(2 0 0)	48.650	1.8700	0.800	48.049	1.8920
1.0 wt % Fe–TiO_2_	(1 0 1)	25.380	3.5065	1.042	25.281	3.5200
	(0 0 4)	37.750	2.3810	0.902	37.800	2.3780
	(2 0 0)	48.290	1.8831	0.937	48.049	1.8920
10 wt % Fe–TiO_2_	(1 0 1)	25.460	3.4956	1.020	25.281	3.5200
	(0 0 4)	37.980	2.3672	0.929	37.800	2.3780
	(2 0 0)	47.830	1.9001	0.968	48.049	1.8920

**Table 3:** Unit cell parameters and grain sizes of TiO_2_ in TCF with different doping amounts of Fe^3+^.

Sample	Unit Cell Parameters		Grain Size (nm)	
	a = b (Å)	c (Å)	TiO_2_ Anatase (101)	TiO_2_ Anatase (200)
0.1 wt % Fe–TiO_2_	3.740	10.674	8.637	10.777
1.0 wt % Fe–TiO_2_	3.766	9.609	7.728	9.188
10 wt % Fe–TiO_2_	3.800	8.911	7.308	8.878

**Table 4:** Unit cell parameters and grain sizes of TiO_2_ in STCF with differing Fe^3+^ doping.

Sample	Unit Cell Parameters		Grain Size (nm)	
	a = b (Å)	c (Å)	TiO_2_ Anatase (101)	TiO_2_ Anatase (200)
0.1 wt % Fe–SiO_2_–TiO_2_	3.774	9.461	9.007	9.068
0.5 wt % Fe–SiO_2_–TiO_2_	3.782	9.081	9.091	9.579
1.0 wt % Fe–SiO_2_–TiO_2_	3.760	9.522	9.080	8.743
5.0 wt % Fe–SiO_2_–TiO_2_	3.798	8.721	8.980	8.740
10.0 wt % Fe–SiO_2_–TiO_2_	3.796	8.900	8.698	9.812

**Table 5:** Wetting performance of composite films coated on wood with differing Fe^3+^ doping as well as with different immersion times.

	STCF-0	STCF-0.05	STCF-0.1	STCF-0.5	STCF-1	STCF-2.5
4 h	110.7	101	106	101.5	112.1	100.9
6 h	114.2	111.5	107.3	108.1	115.8	113.8
8 h	120.9	121	126.2	124.1	117.6	114.1
